# TRAINING CAREGIVERS TO BE INFECTION PREVENTION SUPERHEROES WITH PROJECT FIRSTLINE

**DOI:** 10.1093/geroni/igae098.2206

**Published:** 2024-12-31

**Authors:** Carolyn Ham, Madyson Schuh, Megan Rogers, Erica Ellis

**Affiliations:** Washington State Department of Health, Olympia, Washington, United States; Washington State Department of Health, Tumwater, Washington, United States; University of Washington, Seattle, Washington, United States; University of Washington Northwest Center for Public Health Practice, Seattle, Washington, United States

## Abstract

Facility-acquired infections in long-term care facilities (LTCF) significantly affect resident’s safety and quality of life. Caregiving staff in LTCF are uniquely positioned to prevent infections by completing infection prevention and control (IPC) activities, including hand hygiene and using personal protective equipment. An IPC Needs Assessment (NA) survey was conducted in 2021 by the Washington State Department of Health (DOH) and University of Washington (UW) with 3,308 frontline healthcare respondents. Respondents from LTCF caregivers indicated a preference for self-paced, asynchronous learning experiences and ranked COVID-19 as the most interesting IPC topic. DOH partnered with UW and the Centers for Disease Control and Prevention to develop six Project Firstline Modules (PFM) on IPC topics. Drawing on adult learning theory principles, training content was developed with a focus on the needs and lived experience of caregivers. Each module was offered in English and Spanish and incorporated an instructional video, interactive scenario, job aid, and evaluation. Between February and December 2023, 589 LTCF caregivers completed modules, comprising 62.93% of all users, with the majority (97.3%) reporting that the information was easily understandable and 88.2% reporting they would be able to apply what they learned to their job. PFM completers reported the highest satisfaction for the module on respiratory droplets, consistent with the NA results. The delivery of PFM successfully provided actionable and accessible IPC training for LTCF caregivers. Future efforts to educate caregivers on IPC should consider their preferences in training modes and topics, and further explore public health and academic partnerships.

